# Automation-Induced Complacency Potential: Development and Validation of a New Scale

**DOI:** 10.3389/fpsyg.2019.00225

**Published:** 2019-02-19

**Authors:** Stephanie M. Merritt, Alicia Ako-Brew, William J. Bryant, Amy Staley, Michael McKenna, Austin Leone, Lei Shirase

**Affiliations:** Department of Psychological Sciences, University of Missouri–St. Louis, St. Louis, MO, United States

**Keywords:** automation, complacency, trust, complacency potential, measure, scale

## Abstract

Complacency, or sub-optimal monitoring of automation performance, has been cited as a contributing factor in numerous major transportation and medical incidents. Researchers are working to identify individual differences that correlate with complacency as one strategy for preventing complacency-related accidents. *Automation-induced complacency potential* is an individual difference reflecting a general tendency to be complacent across a wide variety of situations which is similar to, but distinct from trust. Accurately assessing complacency potential may improve our ability to predict and prevent complacency in safety-critical occupations. Much past research has employed an existing measure of complacency potential. However, in the 25 years since that scale was published, our conceptual understanding of complacency itself has evolved, and we propose that an updated scale of complacency potential is needed. The goal of the present study was to develop, and provide initial validation evidence for, a new measure of automation-induced complacency potential that parallels the current conceptualization of complacency. In a sample of 475 online respondents, we tested 10 new items and found that they clustered into two separate scales: *Alleviating Workload* (which focuses on attitudes about the use of automation to ease workloads) and *Monitoring* (which focuses on attitudes toward monitoring of automation). Alleviating workload correlated moderately with the existing complacency potential rating scale, while monitoring did not. Further, both the alleviating workload and monitoring scales showed discriminant validity from the previous complacency potential scale and from similar constructs, such as propensity to trust. In an initial examination of criterion-related validity, only the monitoring-focused scale had a significant relationship with hypothetical complacency (*r* = -0.42, *p* < 0.01), and it had significant incremental validity over and above all other individual difference measures in the study. These results suggest that our new monitoring-related items have potential for use as a measure of automation-induced complacency potential and, compared with similar scales, this new measure may have unique value.

## Introduction

Automation, or the mechanization of processes and tasks formerly carried out by humans, is nearly ubiquitous and has helped to improve the efficiency and safety of a variety of tasks by reducing human error in high-stakes situations (e.g., Parasuraman and Riley, 1997; Hoc et al., 2009; Merritt et al., 2012). However, automation is imperfect, and many operators have moved from active participants in the task to more passive monitors of automation performance (Sheridan, 1987; Bahner et al., 2008). Investigations of several major aviation incidents suggest that one contributing factor is pilot *complacency*, or the failure to adequately monitor the performance of an automated system. Pilots who become complacent may fail to quickly correct automation failures, contributing to major incidents (e.g., Wiener, 1981; Hurst and Hurst, 1982; Casey, 1998; Funk et al., 1999). Complacency is a critical topic in automation safety and has been identified as one of the top five issues related to cockpit automation (Funk et al., 1999). In helping to understand when and why complacency occurs, researchers have suggested that some individuals may have a greater inclination toward complacency than others – an individual difference labeled *automation-induced complacency potential* (i.e., complacency potential).

Several studies have examined the role of complacency potential, in many cases finding correlations between complacency potential and outcomes such as task performance and error detection. To date, the majority of this research has been conducted using Singh et al. (1993) complacency potential scale. While this work has been undoubtedly beneficial, we suggest that after 25 years, the time has come to create an updated scale of complacency potential. Our primary rationale is that major theoretical advances have been made in the conceptualization of automation-induced complacency in recent years. Others include the inconsistencies in factor structures found for the original scale and the issue that several items in the original scale refer to technologies that are no longer in widespread use. Thus, the goal of the present work is to develop a self-report scale of complacency potential that is consistent with the current theory on complacency.

### Automation-Induced Complacency

Definitions of complacency vary, but they generally agree that complacency is evident when (a) there is a human operator monitoring an automated system, (b) the frequency of monitoring behavior is suboptimal or below a normative rate, and (c) suboptimal monitoring leads to performance failures (Parasuraman and Manzey, 2010). Typically, these failures result from the combination of an automation failure with an insufficient response by the operator and may include errors of omission (failure to act on an accurate signal), errors of commission (overreliance on flawed systems or failure to detect a misleading signal), and delayed responses (Parasuraman and Manzey, 2010). Complacency can lead to tragic outcomes in high-risk and high-stakes roles, and complacency is observed in both experts and novice operators of automation (e.g., Singh et al., 1998; Galster and Parasuraman, 2001; Metzger and Parasuraman, 2005).

Often, researchers have operationalized complacency as either a complete failure to detect or as an unacceptably slow response time in detecting the error (e.g., De Waard et al., 1999). Research suggests that complacency is more likely when operators work with highly reliable systems (e.g., Parasuraman et al., 1993; Singh et al., 1997; Bagheri and Jamieson, 2004a), particularly when no explanation for the aid’s behavior is provided (Bagheri and Jamieson, 2004b). May et al. (1993) varied reliability and found people detected more failures when reliability was lower, but even at low levels of reliability, detection of errors was worse than in manual performance. Further, Bailey and Scerbo (2007) found negative relationships between subjective trust and effective monitoring.

However, these effects may only occur when manual and automated workloads compete for the operator’s attention (Parasuraman and Manzey, 2010). Parasuraman et al. (1993) found that when operators were required to multitask, complacency levels varied with automation reliability (complacency was greatest for automated aids with high and constant reliability). However, when the operator had only a single task to perform, complacency was unaffected by automation reliability. In their review, Parasuraman and Manzey (2010) stated that “The operator’s attention allocation strategy appears to favor his or her manual tasks as opposed to the automated task” (p. 387). This suggests that, given a combination of manual and automation tasks, complacency involves *the shifting of attention toward the manual task*. They point out that such a strategy may be rational, particularly when the aid’s reliability is high. However, this attention shift may delay or prevent the identification of aid errors. Some have argued that complacency can only be inferred when an error has occurred; however, this seems somewhat circular in that the construct is defined in part by its assumed outcome (e.g., Moray and Inagaki, 2000; Moray, 2003; Bahner et al., 2008). Others suggest that instead, complacency should be defined by a comparison of the user’s monitoring rate with an “optimal” monitoring rate (e.g., Bahner et al., 2008).

In line with the conceptualization of complacency as an attention shift, several researchers have employed eye tracking studies to examine the extent to which users watched the indicators of automation performance. Metzger and Parasuraman (2005) found that operators classified as complacent (i.e., missing automation errors) looked at the screen area reflecting the automated process less than non-complacent operators. Similar results were found by Bagheri and Jamieson (2004a) and Wickens et al. (2005). Duley et al. (1997) and Singh et al. (1997) attempted to eliminate complacency by centrally locating the automation monitoring task on the screen, but they were unsuccessful. These results suggest that complacency seems to result not simply from visual fixation but from allocation of attention away from the automated task and toward manual tasks.

In summary, the degree of *attention devoted to monitoring automated tasks* (specifically, the lack thereof) seems to be at the core of our current understanding of complacency. Characteristics of the automation (i.e., reliability), the user (i.e., subjective trust), and the situation (i.e., workload) seem to play important roles in producing complacent behavior to the degree that they shift the operator’s attention either toward or away from monitoring the automation’s performance.

### Complacency Potential Rating Scale

Singh et al. (1993) developed the automation induced complacency potential rating scale (CPRS). It is interesting to note that few researchers have offered a specific definition of the construct of complacency potential, but it seems to be generally regarded as an individual difference indicating one’s *propensity to engage in suboptimal monitoring of automation* (e.g., Prinzel et al., 2001; Parasuraman and Manzey, 2010). In their paper, Singh et al. (1993) offered that their scale measures positive attitudes toward automation, which may then create “premature cognitive commitment” (Langer, 1989). Singh et al. (1993) compiled an initial pool of 100 items assessing attitudes toward automation, which was narrowed to a pool of 16 by four readers familiar with the concept of complacency (as it was understood at that time). The items are on a five-point scale ranging from 1 (strongly disagree) to 5 (strongly agree). The primary focus of the CPRS seems to be on trust in various forms of automation, including assessments of relative reliability between automated and human assistants. Example items include, “Automated systems used in modern aircraft, such as the automatic landing system, have made air journeys safer.” and “I feel safer depositing my money at an ATM than with a human teller.” However, given the recent conceptualization of complacent behavior as a failure to monitor optimally (e.g., Parasuraman and Manzey, 2010; Mahfield et al., 2011), we will argue that it may be more effective to create items concerning attitudes toward monitoring automation under conditions of high workload. We will return to this point later.

Singh et al. (1993) performed an exploratory factor analysis on their scale which revealed that the items sorted into four factors which they labeled: confidence (α = 0.82), reliance (α = 0.85), trust (α = 0.89), and safety (α = 0.95). However, it seems relatively common for researchers to compute a total scale score collapsing across the four factors – in other words, the scale is often treated as unidimensional despite the notion that it consists of four factors (e.g., Prinzel et al., 2005; Merritt and Ilgen, 2008; Giesemann, 2013; Leidheiser and Pak, 2014; Mirchi et al., 2015; Pop et al., 2015; Shah and Bliss, 2017). Other researchers have examined the factors separately but have found few clear distinctions among their effects [e.g., Hitt et al., 1999; Kozon et al., 2012 (adapted scale); Verma et al., 2013]. In summary, our literature review suggests variability in how the CPRS’s factor structure has been treated in subsequent research.

We noted that few studies have attempted to replicate the findings of the four-factor structure using factor analysis. One potential reason for this is that factor analysis requires a large sample size, and many CPRS studies had relatively small sample sizes. Indeed, the original factor analysis was performed on a sample of *N* = 139, reflecting a subject-to-item ratio of approximately 7:1. Costello and Osborne (2005) found that factor analyses with a subject-to-item ratio of 5:1 produced the correct factor structure only 40% of the time, while accuracy increased to 60% for ratios of 10:1 and 70% for ratios of 20:1. One of the goals of the present study is to re-evaluate the factor structure of the CPRS using a larger sample size in addition to creating a new measure of complacency potential that reflects our updated understanding of complacency.

### AICP-R Measure Development

Based on our literature review of the nature of complacency, the authors generated a set of items focused on workload and attention to monitoring. This process was primarily undertaken by the first author, who is an expert in the human-automation trust literature. Although no specific theoretical model of complacency seemed to dominate in the literature, items were produced that reflected the major content of the current literature on complacency as reviewed previously (e.g., a focus on attentional processes in multi-task scenarios). Item generation concluded when the item content was deemed to become redundant. Because the construct of complacency (defined as directing attention toward vs. away from automated processes) is relatively narrow, the items became redundant relatively quickly. Thus, our item pool consisted of 10 items. All 10 items were used in the analyses conducted in this study.

Given the importance of workload in complacency, it seems helpful to acknowledge workload in the measure items so that respondents have the appropriate frame of reference (i.e., requirement to multi-task) when responding to the items. Many of the items are situated specifically in terms of high workload or multitasking. Without this element, we suspected that there would likely be restricted variance because, in the absence of a reason not to monitor, it seemed likely that all respondents would indicate that they would monitor frequently. The specification of high workload situations conveys that respondents need to prioritize and balance various responsibilities and is consistent with research indicating that complacency rates are higher under conditions that require divided attention (see Parasuraman and Manzey, 2010). We label our new set of items as the automation induced complacency potential-revised scale (AICP-R).

One additional distinction between these new items and the CPRS is that that these items do not refer to specific technologies (e.g., VCRs or manual card catalogs; Singh et al., 1993). In making this adjustment, our aims were threefold. First, we hoped to decrease item-specific variance in responses. For example, if respondents had no experience with a particular technology referenced by an item, the scale’s internal consistency and validity could be adversely affected. Secondly, given the high rate of technology development, technologies can quickly become outdated. By making generic references to automation, we hope that the scale will better maintain relevance and validity as specific technologies come and go. Thirdly, as automation becomes more prevalent, a large number of tech-specific items would be needed to cover the construct domain. Generic items keep the scale brief while allowing respondents to consider the technologies most familiar to them.

### Convergent/Discriminant Validity

When developing a measure, correlations are used to examine convergent validity (the measure shows expected relationships with other constructs) and discriminant validity (the measure is different from similar constructs; e.g., Clark and Watson, 1995). In the present study, we selected four measures with which to examine correlations with our new scale, as described below. Three of these, propensity to trust machines, perfect automation schema, and preference for multitasking, were selected in order to provide evidence of discriminant validity. Propensity to trust machines and perfect automation schema have been examined in the trust in automation literature and have shown to be substantially distinct from each other (Merritt et al., 2015). To our knowledge, preference for multitasking has not yet been examined in the context of automation use but was included here due to its relevance to attention allocation.

#### CPRS

We will examine correlations with Singh et al. (1993) CPRS scale. We expect moderate positive correlations between the new items and the original CPRS given that they both assess attitudes toward automation. However, we do not expect the correlations to be high because the scales’ foci are slightly different. Whereas the CPRS focuses on trust and perceived reliability of various specific automated systems, the new items focus on attitudes about using and monitoring automation under high workload.

#### Propensity to Trust Machines

Current understandings of complacency acknowledge that complacency is likely related to trust – all else being equal, those who trust a system more will be more likely to become complacent. This relationship between trust and complacency has been seen in some past work (Bailey and Scerbo, 2007). *Propensity to trust machines* refers to a stable, trait-like tendency to trust or not trust machines, including automated systems (Merritt and Ilgen, 2008). We expect to see a positive correlation between propensity to trust machines and complacency potential; however, we also expect discriminant validity. Propensity to trust may correlate with the aspects of complacency potential that reflect a tendency to trust, but research has established the importance of workload concerns. Attention may be diverted from monitoring because of other workload pressures even when trust is low. Thus, we expect some, but not large, overlap between propensity to trust machines and the new complacency potential scale.

#### Perfect Automation Schema

The “perfect automation schema” refers to a tendency to view automation as perfectly performing or infallible. This construct was developed by Dzindolet et al. (2002), who observed that users with higher perfect automation schemas demonstrated greater declines in trust following automation errors, compared to users with lower perfect automation schemas. Based on a review of the literature, Merritt et al. (2015) developed a two-factor measure with factors reflecting *high expectations* for automation performance and *all-or-none beliefs* about automation performance, the latter referring to a belief that automation either works perfectly or not at all. They found low correlations between these two factors, suggesting that they are two separate constructs rather than two elements of the same construct. Only all-or-none beliefs significantly predicted trust declines following automation errors, but in this study, we include both high expectations and all-or-none beliefs in order to examine their convergent and discriminant validity of these constructs with complacency potential, as both of these constructs may have substantial overlap with complacency potential.

#### Preference for Multi-Tasking

As previously discussed, workload is a key factor in complacency (Parasuraman and Manzey, 2010) such that users direct their attention away from the automated task and toward other, manual tasks, whereas complacency is less likely to be observed under single-task conditions. Multi-tasking is defined as “the ability to integrate, interleave, and perform multiple tasks and/or component subtasks of a larger complex task” (Salvucci et al., 2004, p. 267). A preference for multi-tasking, then, is one’s preference for performing multiple tasks simultaneously (Poposki and Oswald, 2010). We examine correlations between complacency potential and preference for multi-tasking (also known as *polychronicity*) to ensure that our assessment of complacency potential does not fully overlap with this previously existing construct, preference for multi-tasking.

Research Question 1: To what extent does the AICP-R demonstrate convergent or discriminant validity with (a) CPRS, (b) propensity to trust machines, (c) perfect automation schema, and (d) preference for multi-tasking?

### Criterion-Related Validity

We also seek to provide some preliminary evidence of the AICP-R’s criterion-related validity. We do so by posing hypothetical scenarios to respondents about monitoring various types of automation under high workload and varying risk levels. Respondents are asked to indicate how often they would monitor the automated system in each scenario. Lower levels of monitoring are interpreted as higher complacency. Although obtaining actual monitoring behavior using techniques such as eye tracking will provide stronger evidence than hypothetical scenarios, such techniques were beyond the scope of the present study and are suggested as an avenue for future research. In the meantime, we obtain preliminary evidence of criterion-related validity by examining whether the AICP-R has incremental validity over and above the CPRS in predicting hypothetical complacency.

Research Question 2: Does the AICP-R have incremental validity beyond the CPRS in predicting hypothetical complacency?

## Materials and Methods

### Participants and Data Cleaning

Five hundred workers from Amazon Mechanical Turk (MTurk) participated in this study. MTurk is an online crowdsourcing platform where individuals can register to complete brief tasks, including research studies, for a small amount of compensation. Participants responded to 60 items and were compensated with $0.50. To maximize data quality, insufficient effort responding metrics were employed (see Huang et al., 2012, for more information on these metrics). These included long string, individual reliability, and psychometric antonyms analyses. Sixteen individuals were flagged by at least two of the three indicators and their data were removed. Using Mahalanobis distances, nine additional participants were flagged as multivariate outliers and, after visual inspection of their data, they were subsequently removed, totaling 25 participants removed from the dataset.

The retained sample of *N* = 475 was 54.5% male, 45.3% female, and.2% gender non-conforming. The age range of subjects was 19–75 years with a mean of 35.39. With regards to ethnicity, 6.5% of the sample identified as Hispanic while 93.5% identified as Non-Hispanic. The sample consisted of Caucasian (78.1%), African American (6.9%), East Asian/Pacific Islander (6.1%), Latino/Latina (4.6%), multi-racial (2.1%), South Asian (1.1%), Native American/Alaskan Native (0.6%), and other (0.4%) respondents.

### Procedure

After providing consent, respondents completed a web-based survey beginning with demographic and descriptive items. The respondents then completed the study measures and provided ratings on the hypothetical complacent behavior scale.

### Measures

#### Propensity to Trust Machines

The tendency to generally trust machines was examined using the Propensity to Trust Machines questionnaire which was developed by Merritt (2011). This scale consists of six items with response options ranging from 1 (*strongly disagree*) to 5 (*strongly agree*). Example items include, “I usually trust machines until there is a reason not to,” and “For the most part, I distrust machines.”

#### Complacency Potential Rating Scale (CPRS)

The original CPRS consisted of 16 items (Singh et al., 1993). However, their publication lists only 12 of these and we were unable to obtain the other four. Therefore, we included those 12 items with response options on a five-point Likert scale ranging from 1 (*strongly agree*) to 5 (*strongly disagree*). Example items include, “I think that automated devices used in medicine, such as CT scans and ultrasound, provide very reliable medical diagnosis” and “Automated devices in medicine save time and money in the diagnosis and treatment of disease.”

#### Perfect Automation Schema

This is a 10-item measure developed by Merritt et al. (2015). It is divided into two subscales: high expectations, which measures participants’ expectations of automation performance and all-or-none thinking, which classifies participants based on their tendency to assume a machine to be broken if it doesn’t function properly. The responses for this scale were also scored on a five-point Likert type scale which ranged from 1 (*strongly disagree*) to 5 (*strongly agree*). An example item from the high expectations scale is, “Automated systems can always be counted on to make accurate decisions.” An example from the all-or-none thinking scale is, “If an automated system makes an error, then it is broken.”

#### Multitasking Preference Inventory

This is a modified 5-item scale (originally 14 items) that assesses one’s preference for multitasking. It was developed by Poposki and Oswald (2010). Example items include “When doing a number of assignments, I like to switch back and forth between them rather than do one at a time” and “When I have a task to complete, I like to break it up by switching to other tasks intermittently.” In order to limit the length of this study, and because this relationship was less established than the others to be examined, the five items with the highest factor loadings reported by Poposki and Oswald (2010) were utilized.

#### Hypothetical Complacent Behavior

This scale was developed by the authors for use in this study. This is a 5-item scale with a five-point Likert response format ranging from 1 (*Never*) to 5 (*Constantly*)^1^. It asks participants to indicate their anticipated level of monitoring in hypothetical situations that involve automation. Because past research has determined that complacent behavior only occurs in multi-tasking situations, the instructions specified that respondents should imagine that they are very busy and have a lot to do. Example items include “If I were riding in a self-driving car” and “If I were using auto-pilot in a passenger jet full of people.”

## Results

### Descriptive Statistics and Confirmatory Factor Analyses

Before analyzing the complacency potential scales, we first verified the factor structures of the scales used to test convergent and discriminant validity. Scale means, standard deviations, reliabilities, distributional properties, and the fit statistics of confirmatory factor analyses for (a) propensity to trust, (b) the two perfect automation schema scales, and (c) preference for multitasking are presented in Table 1. Because our focus of the study is the development of a new AICP scale and comparison with the existing scale, the complacency potential scales are treated separately in the following sections. The propensity to trust and preference for multitasking scales demonstrated good psychometric properties, with well-fitting unidimensional models and acceptable reliabilities.

**Table 1:** Means, standard deviations, internal consistencies, distributional properties, and fit of confirmatory factor analysis for propensity to trust, perfect automation schema scales (high expectations and all-or-nothing thinking), and multitasking preference.

Scale	*N*	*M*	*SD*	a	Skew	Kurtosis	χ^2^_(df)_	RMSEA	CFI	TLI
Propensity to trust	475	3.92	0.65	0.89	-1.00	2.70	45.34_(9_)	0.09	0.98	0.96
Perfect automation schema	475						111.14_(13)	0.13	0.86	0.77
High expectations	475	2.65	0.68	0.69	0.25	0.48				
All-or-nothing thinking	475	2.70	0.75	0.64	0.26	-0.24				
Multitasking preference	475	2.93	1.02	0.93	0.08	-1.03	1.11_(5)	0.00	1.00	1.00

*Statistics shown for 4-item high expectations scale and 3-item all-or-nothing thinking scale (consistent with Merritt et al., 2015). CFA statistics reflect the fit of the final model for each scale (tested individually)*.

In regard to the perfect automation schema scales, we replicated the findings of Merritt et al. (2015) that the reverse-worded items had poor psychometric properties. So, consistent with Merritt et al. (2015), we dropped those items. Nevertheless, the fit of this two factor model was poor and substantially less well-fitting than in Merritt et al.’s (2015) sample, and the internal consistencies for each subscale were inadequate. Further, while Merritt et al. (2015) found relatively low correlations between the two scales in their two samples (Φs = 0.05 and 0.38, respectively), our correlation was much higher (Φ = 0.60, *p* < 0.01). Thus, while the psychometric properties for propensity to trust and multitasking preference generalized well from past research, the perfect automation schema scales did not.

### AICP-R Scale Descriptive Information and Dimensionality

Descriptive information for each AICP item (Table 2) and inter-item correlations (Table 3) were examined. All items were judged to have adequate variances (SDs > 0.66) with most items showing the full range of responses. Table 3 shows the inter-item correlations. Many of them were significant and moderate in magnitude; however, a visual examination suggests that multiple factors may be present. This possibility is further explored in the factor analysis to follow.

**Table 2:** AICP-R item content and descriptive statistics.

	Item	Mean	SD	Min	Max	Skewness	Kurtosis	Item-Total *r*
1	When I have a lot to do, it makes sense to delegate a task to automation.	4.02	0.72	1	5	-0.92	4.93	0.47
2	If life were busy, I would let an automated system handle some tasks for me.	4.09	0.73	1	5	-0.86	4.44	0.42
3	Automation should be used to ease people’s workload.	4.22	0.66	2	5	-0.57	3.58	0.39
4	If automation is available to help me with something, it makes sense for me to pay more attention to my other tasks.	4.02	0.71	1	5	-0.64	3.97	0.43
5*^r^*	Even if an automated aid can help me with a task, I should pay attention to its performance. [R]	1.99	0.71	1	5	0.83	4.73	0.24
6	Distractions and interruptions are less of a problem for me when I have an automated system to cover some of the work.	3.82	0.79	1	5	-0.77	3.75	0.39
7	Constantly monitoring an automated system’s performance is a waste of time.	2.82	1.03	1	5	0.32	2.25	0.44
8*^r^*	Even when I have a lot to do, I am likely to watch automation carefully for errors. [R]	2.75	0.99	1	5	0.12	2.16	0.44
9	It’s not usually necessary to pay much attention to automation when it is running.	3.05	1.03	1	5	-0.11	1.98	0.54
10	Carefully watching automation takes time away from more important or interesting things.	3.44	0.96	1	5	-0.45	2.50	0.45

*^*r*^Recoded items post-recode. N = 475.*

**Table 3:** AICP-R scale inter-item correlations.

	1	2	3	4	5	6	7	8	9	10
1	1.00									
2	0.66**	1.00								
3	0.48**	0.55**	1.00							
4	0.55**	0.53**	0.48**	1.00						
5^r^	0.01	-0.04	-0.02	-0.05	1.00					
6	0.52**	0.46**	0.40**	0.45**	-0.04	1.00				
7	0.10*	0.04	0.08	0.08	0.28**	0.10*	1.00			
8^r^	0.12**	0.06	0.04	0.07	0.41**	0.06	0.41**	1.00		
9	0.13**	0.12*	0.16**	0.14**	0.28**	0.19**	0.48**	0.49**	1.00	
10	0.10*	0.05	0.05	0.16**	0.22**	0.07	0.46**	0.41**	0.53**	1.00

*^∗^Represents significance at the 0.05 level. ^∗∗^Represents significance at the 0.01 level. ^*r*^Recoded items. Coefficients represent post-recode correlations. N = 475.*

In order to determine the factor structure of AICP-R, exploratory (EFA) and confirmatory (CFA) factor analyses were conducted. Our sample was split into two random halves; EFA was performed on sample 1 to establish a factor structure, and that structure was confirmed using CFA in sample 2.

**Figure 1:**
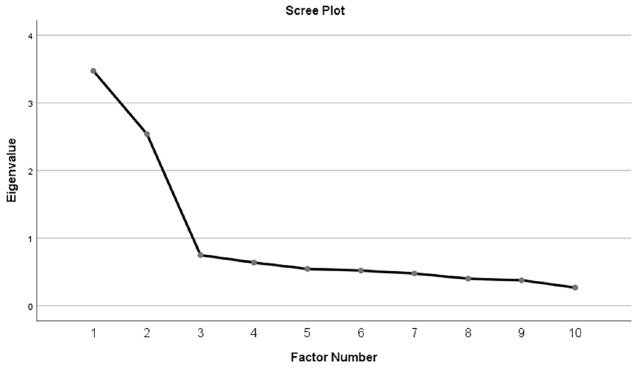
Scree plot for exploratory factor analysis on AICP-R scale.

For the EFA, scree plots using parallel analysis were first generated, which suggested a two factor solution (see Figure 1). Principal axis factoring and oblimin rotation were utilized to account for the possibility that the factors could be significantly correlated. Two factors emerged (factor loadings are found in Table 4). An analysis of the item content for each factor suggests that Factor 1 focuses on the use of automation to relieve high workload (e.g., “Automation should be used to ease people’s workload”). Thus, we label this factor “*Alleviating Workload* (AW).” The items loading on Factor 2 focus on the monitoring, rather than the use, of automation (“It’s not usually necessary to pay much attention to automation when it is running”); thus we label Factor 2 *“Monitoring* (M)*.”* Using this split half sample, scale reliability estimates for each factor suggested acceptable consistencies for each factor(α_AW_ = 0.87, α_M_ = 0.79).

**Table 4:** AICP-R factor loadings (exploratory factor analysis).

Factor:	1	2
Factor label:	AW	M
Item 1	0.77	
Item 2	0.78	
Item 3	0.68	
Item 4	0.72	
Item 5		0.49
Item 6	0.63	
Item 7		0.54
Item 8		0.63
Item 9		0.67
Item 10		0.59

*Factors 1 and 2 correlate at 0.12. EFA performed with principal axis factoring and direct oblimin rotation. AW, alleviating workload; M, monitoring. N = 239.*

Next, CFA was used to confirm the fit of the two factor solution using the other split-half sample (*N* = 236). Results suggested that the two factor model was a good fit for the data (χ^2^ = 84.33, *df* = 34) and was a significant improvement over the fit of a unidimensional model (χ^2^ = 317.97; Δχ^2^ = 233.64, Δdf = 1; *p* < 0.01). Additional fit indices for the two-factor specification include the Comparative Fit Index (*CFI*; Hu and Bentler, 1999) the Tucker-Lewis Index (*TLI*; Tucker and Lewis, 1973), and the root mean square error of approximation (*RMSEA*; Steiger, 1990). All of these indices suggested adequate fit of the two-factor model to the data [RMSEA = 0.08 (0.06, 0.10); CFI = 0.92; TLI = 0.89]. The latent factor correlation between alleviating workload and monitoring (corrected for unreliability) was only F = 0.21. Given this low correlation, we believe that alleviating workload and monitoring should be treated as two separate scales rather than two subfactors of the same construct.

In summary, our analyses of the dimensionality of our 10 new items suggested that scales measuring attitudes about (a) the use of automation to ease workload and (b) frequency of monitoring under high workload conditions should be considered distinct.

### CPRS Dimensionality

To assess the factor-structure of the CPRS, a CFA was conducted on these data using the lavaan package (Rosseel, 2012) in R (R Core Team, 2016). To be consistent with the scale’s original development, a four-factor solution was tested first, following the same item-factor relationships indicated in Singh et al.’s (1993) original publication. Heywood cases (negative error variances) were obtained for items 9 and 12, so these two error variances were fixed to zero. The results indicated a poor fit of the four factor model to the data (χ^2^ = 408.31, df = 51, *p* < 0.01; RMSEA = 0.12 (0.11, 0.13); CFI = 0.71; TLI = 0.62). An examination of the modification indices suggested that fit would be improved by allowing several items to cross load on different factors. These results suggest that the original four factor solution identified by Singh et al. (1993) did not generalize well to this sample.

Given that many researchers have combined all of the items into a composite score (essentially treating the scale as unidimensional), we also tested the fit of a unidimensional model. In this case, it was not necessary to fix any error variances, so they were all freely estimated. This model also produced a poor fit to the data [χ^2^ = 349.53, df = 54, *p* < 0.01; RMSEA = 0.11 (0.10, 0.12); CFI = 0.76; TLI = 0.71], with item 11 having a non-significant loading on the latent factor (l = 0.14, *p* = 0.21).

In summary, neither the hypothesized four factor CPRS model, nor the unidimensional CPRS model, fit our data well. Our recommendation to researchers wishing to use the CPRS in subsequent research is to carefully examine the inter-item relationships, with large sample sizes, to provide additional insight into how the scale items should be treated. Data files and output for these analyses can be found in the Supplementary Data Sheet S1.

### Relationship Between CPRS and AICP-R

Internal consistency reliabilities and correlations among alleviating workload, monitoring, and the four CPRS factors are found in Table 5. Alphas were well below desirable levels for the CPRS reliance, trust, and safety subfactors. Interestingly, results indicate that the alleviating workload scale was moderately and significantly associated with each of the four CPRS subfactors (*rs* = 0.29–0.58). However, the relationships between monitoring and the four CPRS factors were substantially lower (*rs* = 0.01–0.11). Thus, it seems that the item content of the alleviating workload scale has greater conceptual and empirical commonalities with the CPRS scale than does the monitoring scale.

**Table 5:** CPRS and AICP-R subscale reliabilities and correlations.

	1	2	3	4	5	6
1. Alleviating workload	(0.84)					
2. Monitoring	0.14**	(0.77)				
3. CPRS confidence	0.58**	0.05	(0.71)			
4. CPRS reliance	0.47**	0.01	0.54**	(0.39)		
5. CPRS trust	0.48**	0.08	0.44**	0.48**	(0.38)	
6. CPRS safety	0.29**	0.11*	0.23**	0.18**	0.19**	(0.34)

*^∗^Represents significance at the 0.05 level. ^∗∗^Represents significance at the 0.01 level. Values on the diagonal line represent the Cronbach’s alpha reliability coefficient for the respective scale. N = 475.*

### AICP-R Discriminant Validity

In order to conclude that our automated complacency scale was distinct from theoretically similar scales, several analyses were conducted to assess AICP-R’s discriminant validity. Scales used for discriminant validity analyses were the Perfect Automation Schema scale (Merritt et al., 2015), the Propensity to Trust Machines scale (Merritt, 2011), and the Multi-Tasking Preference Inventory (Poposki and Oswald, 2010). Correlations of all scales can be seen in Table 6. Correlations with the CPRS are also displayed for reference.

**Table 6:** Correlations for convergent/discriminant validity.

		1	2	3	4	5	6	7	8
	Mean	3.56	4.03	3.00	2.70	2.65	3.92	2.93	3.75
	SD	0.39	0.59	0.60	0.75	0.67	0.65	1.02	0.78
1	CPRS	(0.74)							
2	Allev. workload	0.54**	(0.84)						
3	Monitoring	0.05	0.14**	(0.77)					
4	All-or-none	0.11*	-0.02	0.05	(0.64)				
5	High expect.	0.38**	0.25**	0.26**	0.38**	(0.69)			
6	Propensity to t.	0.54**	0.56**	0.16**	-0.04	0.35**	(0.89)		
7	Multi-tasking	0.14**	0.10*	0.08	0.03	0.11*	0.09*	(0.93)	
8	Complacency	-0.08	-0.07	-0.38**	-0.09	-0.25**	-0.15**	-0.00	(0.82)

*^∗^Represents significance at the 0.05 level. ^∗∗^Represents significance at the 0.01 level. N = 475. Allev. workload, alleviating workload scale; High expect., high expectations of the PAS scale; Propensity to T., propensity to trust machines scale; Multi-tasking, preference for multitasking scale; Complacency, hypothetical complacent behavior scale*.

The CPRS and the alleviating workload scale exhibited moderate and similar correlations with propensity to trust machines (*rs* = 0.54 and 0.56, respectively), indicating these scales tap an element of propensity to trust, but they do not overlap so substantially that they could be considered the same construct. The correlations of these two scales (CPRS and alleviating workload) were also similar in magnitude with high expectations and preference for multitasking. Thus, these correlations also suggest that the CPRS and alleviating workload may tap similar content.

In contrast, monitoring demonstrated far less overlap with propensity to trust machines, suggesting that perhaps one’s willingness to monitor under high workload conditions relates little to one’s stable propensity to trust. Monitoring did correlate moderately with high expectations for automation performance, but had non-significant correlations with all-or-nothing thinking and with preference for multitasking.

As an exploratory analysis, we conducted a confirmatory factor analysis using alleviating workload, monitoring, the unidimensional CPRS, the two PAS scales, propensity to trust, and preference for multitasking. All latent variables were intercorrelated. This model showed adequate fit [χ^2^_(719)_ = 2087.15, RMSEA = 0.06 (0.06, 0.07), CFI = 0.84, TLI = 0.82]. Based on a visual inspection of the correlations, an alternative model was proposed in which propensity to trust was a higher order factor upon which the other scales loaded. Alleviating workload, high expectations, and the CPRS had relatively higher factor loadings on the higher order propensity to trust factor, but monitoring, all-or-nothing thinking, and preference for multitasking did not. Overall, the fit of this model was not as strong as the model treating the factors as separate [χ^2^_(734)_ = 2324.31, RMSEA = 0.07 (0.06, 0.07), CFI = 0.81, TLI = 0.80]. A second alternative model having only AW, HE, and CPRS loading on a higher-order propensity to trust factor, with the remaining latent factors correlated, also did not fit as well as the original model with separate scales [χ^2^_(731)_ = 2319.94, RMSEA = 0.07 (0.07, 0.07), CFI = 0.81, TLI = 0.80].

### Initial Criterion-Related Validity Evidence

To provide some preliminary evidence regarding the incremental validity of the new AICP-R factors over the CPRS, hierarchical linear regression analyses were conducted. Hypothetical complacency was regressed on the CPRS in Step 1, and the two AICP-R scales were added in Step 2. The direct relationship of the CPRS with hypothetical complacency was approaching significance (*B* = -0.17, *p* = 0.07). Adding the new AICP-R factors in Step 2 produced a significant increase in *R*^2^ (see Table 7). An examination of the beta coefficients reveals that alleviating workload was not significantly related with complacency (*B* = 0.03, *n.s.*). However, monitoring was significantly and negatively associated with complacency (*B* = -0.42, *p* < 0.01), such that those who scored higher on the monitoring scale were less complacent. In other words, the monitoring scale provided unique variance to the prediction of hypothetical complacency.

**Table 7:** Results of hierarchical regression analysis predicting hypothetical complacency.

	Hypothetical complacency	
	Model 1	Model 2
CPRS	-0.17	
Alleviating Workload		0.03
Monitoring		-0.42**
Adjusted *R*^2^	0.01	0.14
Δ*F*		38.19
Δ*R*^2^		0.14**

*Unstandardized betas displayed. N = 475. ^∗^p < 0.05; ^∗∗^*p* < 0.01*.

As an even stronger test, we performed a similar regression in which all of the pre-existing scales, including the CPRS, propensity to trust, high expectations, all-or-nothing thinking, and multitasking preferences were entered in block 1 (results displayed in Table 8). Of these, only high expectations had a significant relationship with complacency (*B* = -0.27, *p* < 0.01), although propensity to trust was approaching significance (*B* = -0.12, *p* = 0.08). Alleviating workload had no incremental validity above the scales in block 1 (*B* = -0.07, *p* = 0.38). However, again, monitoring had significant incremental validity above the other scales, with higher scores on the monitoring scale associating with lower complacency scores (*B* = -0.38, *p* < 0.01).

**Table 8:** Results of hierarchical regression analysis predicting hypothetical complacency with all study scales.

	Hypothetical complacency	
	Model 1	Model 2
CPRS	-0.11	
Propensity to trust	-0.12	
High expectations	-0.27**	
All-or-nothing	-0.01	
Multitasking pref.	0.02	
Alleviating workload		-0.01
Monitoring		-0.38**
Adjusted *R*^2^	0.06	0.16
Δ*R*^2^		0.10**
Δ*F*		28.68

*Unstandardized betas displayed. N = 475. This analysis uses the 4-item high expectations scale and 3-item all-or-nothing thinking scale. ^∗^p < 0.05; ^∗∗^*p* < 0.01*.

## Discussion

Over the past several years, researchers studying automation-induced complacency have focused their conceptualization of complacent behavior on the degree to which users monitor the automated system’s performance. The goal of this research was to develop, and provide some initial validation evidence for, an updated measure of automation induced complacency potential. In doing so, we focused on items that we believe reflect more recent conceptualizations of what complacency is – specifically, a focus on redirection of attention away from monitoring of automation in favor of other tasks.

The results of our analysis suggest that our items formed two potentially different scales. *Alleviating Workload* is relatively similar to the CPRS in that the focus is on attitudes about using automated systems in order to improve performance or ease workload. Consistent with this, we found moderate correlations between these two scales (*r* = 0.54). This supports the contention of Giesemann (2013) that the complacency potential (as traditionally measured) taps attitudes about the use of technologies. However, neither of these two scales significantly correlated with hypothetical complacency behavior.

In contrast, *Monitoring* focused on decisions about whether to direct attention toward monitoring automation performance under conditions of high workload. This scale reflects conceptualizations of complacency by authors including Parasuraman and Manzey (2010). In this sample, the monitoring scale scores were not highly associated with either the CPRS (*r* = 0.05) or alleviating workload (*r* = 0.14), suggesting that decisions about initializing use (alleviating workload) and checking in during performance (monitoring) seem to be empirically distinct. This finding supports the argument that use and reliance decisions should be considered separate, as well as the need to attend to levels of automation (Sheridan and Verplank, 1978; Parasuraman et al., 2000) as interactions at different levels of automation may be influenced by distinct individual differences. The monitoring scale significantly correlated with hypothetical complacency, such that higher scores on this scale were associated with lower preferences to monitor (*B* = -0.42), and this relationship persisted even when all other scales in the study were accounted for (*B* = -0.38). Further, this scale had discriminant validity from similar scales such as propensity to trust machines, the perfect automation schema, and preference for multitasking.

The results suggesting that the monitoring items correlated most strongly with hypothetical complacency is consistent with the recent focus on complacency as a diversion of attention away from monitoring automated performance (Parasuraman and Manzey, 2010). It seems reasonable that if complacency is conceptualized in that way, complacency potential should be defined as one’s propensity to monitor automation sub-optimally, and the monitoring items from this study seem to best represent that definition of complacency potential. Thus, complacency items that focus on broad tendencies to avoid monitoring would reasonably seem to best predict complacent behavior. In comparison, the alleviating workload scale may better predict choices of whether to activate an automated system when given the choice between automated or manual performance.

A secondary contribution of this study was to attempt to replicate the factor structure of the CPRS using a large sample (*N* = 475). We found that both the original four factor structure and a unidimensional structure fit our data poorly. These results suggest that the factor structure of the CPRS may not be invariant across samples, or across time (Vandenberg and Lance, 2000). One potential reason for this is that the scale’s factor structure was originally tested on a relatively small sample size that may have produced unstable results (Costello and Osborne, 2005). Another potential reason is that some of the items refer to technologies that are no longer in widespread use, and this may introduce extraneous variance into the responses for those items. A third potential reason is that while the scale originally consisted of 16 items, our examination included only the 12 that were available to us. Overall, however, our results suggest that the scale’s factor structure may vary across samples or across time.

### Potential Limitations and Suggestions for Future Research

One potential limitation of the current study was that our sample consisted of relatively naïve automation users. Their experience with automation was predominantly with relatively low-stakes and common forms of automation, such as in-car navigation systems. Professionals working in high-stakes settings, such as commercial pilots, may respond differently to these items than do broader samples of respondents. Thus, one suggestion for future research would be to examine the psychometric properties of these items in a specific sample of professionals in safety critical occupations.

Secondly, we suggest that future research examine the relationships between the AICP-R (specifically, the monitoring scale) and actual complacent behavior. Although our self-report, hypothetical complacency scale provided some initial evidence of criterion-related validity, hypothetical behavior does not always correspond with actual behavior. For example, research suggests that while people are moderately risk-averse in hypothetical scenarios, their risk aversion increases as potential costs increase in real scenarios (Holt and Laury, 2002). Therefore people may display higher rates of monitoring in real, high-stakes scenarios, which could produce range restriction in observed relationships. Furthermore, our outcome measure was newly developed, and establishing its relationships with actual complacent behavior would help provide confidence in its validity. Overall, much stronger evidence would be provided via an examination of behavioral outcomes, such as through eye tracking studies or studies designed to assess delay in identifying aid failures. In such studies, we also encourage researchers to include the CPRS and to examine the extent to which the AICP-R (monitoring) has incremental validity beyond the CPRS.

Another direction for future research may be to explore the neural correlates of complacency. Research shows that activation of the brain’s default-mode network is associated with lapses in sustained task attention versus task engagement (e.g., Bonnelle et al., 2011; Ossandon et al., 2011; Pfefferbaum et al., 2011). Others have integrated this research with the study of vigilance performance. Because sustained monitoring of automation can be considered a vigilance task, complacency in the form of failure to monitor may be associated with increased activation in the default-mode network. Thomson et al. (2015) incorporated this perspective into their resource-control theory of vigilance, and Casner and Schooler (2015) found that actual pilots in the cockpit had an almost “irresistible urge to let one’s thoughts drift.” Recent scholars (e.g., Fortenbraugh et al., 2017) have drawn attention to the importance of examining default-mode activation within-persons as well as between-persons. To the extent that some individuals may have chronically greater default-mode activation than others, those individuals may have higher complacency potential. This relationship could be inferred from associations between complacency potential scores and experimental vigilance measures.

In addition, our measure is context-free in that specific technologies are not referenced. This is consistent with the theoretical treatment of complacency potential as spanning various technologies and has the benefit that capturing all of the various automation that individuals may encounter would require a large number of items, and those item would need to be continuously revised as technology evolves. However, the assumption that complacency potential spans targets is currently untested, and future studies might measure complacency potential for specific technologies and assess whether between-person variance is significantly larger than within-person variance in the scores. Further, while keeping the items context-free offers benefits of breadth and brevity, it may mask variation among participants. For example, some individuals may have a stable potential for complacency across various forms of automation, whereas others may vary widely such that they have high complacency in some domains but not in others. Future research might use context-specific measures such as the CPRS to examine variability in complacency potential across situations.

Finally, we suggest that future research continue to identify situational moderators of the relationship between complacency potential and complacent behavior. For example, the link between individual differences and behavior can be affected by variables such as *situation strength*, or the degree to which the situation involves clear scripts or norms for behavior (Meyer et al., 2010). Better understanding the moderators of this relationship will help identify interventions that may buffer individuals with high complacency potential from actually becoming complacent.

## Conclusion

Accurately measuring automation-induced complacency potential can improve performance and safety in a variety of occupations in which automation is employed. Better understanding which operators may have a propensity to become complacent can help target interventions toward those individuals as needed. The present study was an initial step in developing a new measure of individual differences in complacency potential. It reflects current conceptualizations of complacency by focusing on a tendency to monitor automation under high workload. If future research continues to support the validity of this scale, it can be adopted to study complacency and complacency potential.

## Ethics Statement

This study was carried out in accordance with the recommendations of the Institutional Review Board of the University of Missouri–St. Louis with written informed consent from all subjects. All subjects gave written informed consent in accordance with the Declaration of Helsinki. The protocol was approved by the IRB of the University of Missouri–St. Louis.

## Author Contributions

SM generated project idea, obtained IRB approval, collected data, performed analyses, and led the manuscript write-up. AA-B, WB, and AS assisted with data analyses and project write-up. MM and AL both assisted with data analyses and project writeup. LS assisted with project write-up.

## Conflict of Interest Statement

The authors declare that the research was conducted in the absence of any commercial or financial relationships that could be construed as a potential conflict of interest.

## Footnotes

1 This measure originally included five additional items reflecting less-dangerous forms of automation (e.g., an automated vacuum, a spell checker in a word processing program). However, these items were dropped from the final analysis because an Item Response Theory (IRT) analysis using the graded response model with maximum likelihood estimation indicated that they loaded on a separate factor and that the items in that factor had little informational value (scores on those items predominantly seemed to reflect mostly error).
