# Supplementary material for: Automation-Induced Complacency Potential: Development and Validation of a New Scale
Source: Front Psychol. 2019 Feb 19;10:225. doi: 10.3389/fpsyg.2019.00225 (PMC6389673; doi:10.3389/fpsyg.2019.00225)
Supplement: Supplementary file 1 [file Data_Sheet_1.ZIP › Output to submit with revision/Measurement model including all predictors.docx]

| # Measurement model including all predictors  > standardizedSolution(measM)  lhs op rhs est.std se z pvalue  1 AW =~ AICP1 0.802 0.021 38.332 0.000  2 AW =~ AICP2 0.794 0.021 36.992 0.000  3 AW =~ AICP3 0.661 0.029 22.409 0.000  4 AW =~ AICP4 0.682 0.028 24.156 0.000  5 AW =~ AICP6 0.629 0.031 20.154 0.000  6 M =~ AICP5 0.424 0.044 9.735 0.000  7 M =~ AICP7 0.657 0.033 19.628 0.000  8 M =~ AICP8 0.643 0.034 18.853 0.000  9 M =~ AICP9 0.761 0.029 26.273 0.000  10 M =~ AICP10 0.663 0.033 19.958 0.000  11 CPRS =~ CPRS1 0.676 0.029 23.427 0.000  12 CPRS =~ CPRS2 0.645 0.031 21.021 0.000  13 CPRS =~ CPRS3 0.497 0.038 13.067 0.000  14 CPRS =~ CPRS4 0.676 0.029 23.437 0.000  15 CPRS =~ CPRS5 0.434 0.041 10.664 0.000  16 CPRS =~ CPRS6 0.697 0.028 25.253 0.000  17 CPRS =~ CPRS7 0.318 0.045 7.127 0.000  18 CPRS =~ CPRS8 0.362 0.043 8.373 0.000  19 CPRS =~ CPRS9 0.400 0.042 9.529 0.000  20 CPRS =~ CPRS10 0.477 0.039 12.229 0.000  21 CPRS =~ CPRS11 0.112 0.049 2.302 0.021  22 CPRS =~ CPRS12 0.353 0.044 8.093 0.000  23 HE =~ HE1 0.679 0.034 19.687 0.000  24 HE =~ HE2 0.459 0.043 10.558 0.000  25 HE =~ HE3 0.648 0.036 18.131 0.000  26 HE =~ HE6 0.650 0.036 18.262 0.000  27 AN =~ AN1 0.592 0.044 13.604 0.000  28 AN =~ AN2 0.628 0.043 14.651 0.000  29 AN =~ AN4 0.618 0.043 14.354 0.000  30 Prop =~ PT1 0.779 0.021 37.985 0.000  31 Prop =~ PT2 0.656 0.028 23.122 0.000  32 Prop =~ PT3 0.778 0.021 37.884 0.000  33 Prop =~ PT4 0.856 0.015 56.377 0.000  34 Prop =~ PT5 0.847 0.016 53.626 0.000  35 Prop =~ PT6 0.684 0.027 25.634 0.000  36 MT =~ MT1 0.784 0.019 40.800 0.000  37 MT =~ MT2 0.896 0.011 80.971 0.000  38 MT =~ MT3 0.783 0.019 40.497 0.000  39 MT =~ MT4 0.913 0.010 93.024 0.000  40 MT =~ MT5 0.906 0.010 87.808 0.000  **41 AW ~~ M 0.182** 0.054 3.359 0.001  **42 AW ~~ CPRS 0.792** 0.027 29.385 0.000  **43 AW ~~ HE 0.232** 0.056 4.128 0.000  44 **AW ~~ AN -0.065** 0.062 -1.055 0.291  45 **AW ~~ Prop 0.653** 0.033 19.829 0.000  46 **AW ~~ MT 0.111** 0.051 2.204 0.027  47 **M ~~ CPRS 0.080** 0.056 1.428 0.153  48 **M ~~ HE 0.347** 0.056 6.246 0.000  49 **M ~~ AN 0.073** 0.064 1.138 0.255  50 **M ~~ Prop 0.207** 0.052 3.966 0.000  51 **M ~~ MT 0.101** 0.053 1.928 0.054  52 **CPRS ~~ HE 0.206** 0.058 3.570 0.000  53 **CPRS ~~ AN -0.126** 0.062 -2.024 0.043  54 **CPRS ~~ Prop 0.723** 0.029 24.577 0.000  55 **CPRS ~~ MT 0.042** 0.052 0.805 0.421  56 **HE ~~ AN 0.583** 0.054 10.787 0.000  57 **HE ~~ Prop 0.392** 0.050 7.846 0.000  58 **HE ~~ MT 0.147** 0.055 2.684 0.007  59 **AN ~~ Prop -0.038** 0.060 -0.638 0.523  60 **AN ~~ MT 0.061** 0.059 1.033 0.302  61 **Prop ~~ MT 0.101** 0.049 2.049 0.040  62 AICP1 ~~ AICP1 0.356 0.034 10.607 0.000  63 AICP2 ~~ AICP2 0.370 0.034 10.867 0.000  64 AICP3 ~~ AICP3 0.564 0.039 14.471 0.000  65 AICP4 ~~ AICP4 0.535 0.039 13.887 0.000  66 AICP6 ~~ AICP6 0.604 0.039 15.356 0.000  67 AICP5 ~~ AICP5 0.820 0.037 22.225 0.000  68 AICP7 ~~ AICP7 0.569 0.044 12.931 0.000  69 AICP8 ~~ AICP8 0.586 0.044 13.366 0.000  70 AICP9 ~~ AICP9 0.421 0.044 9.564 0.000  71 AICP10 ~~ AICP10 0.561 0.044 12.750 0.000  72 CPRS1 ~~ CPRS1 0.543 0.039 13.905 0.000  73 CPRS2 ~~ CPRS2 0.585 0.040 14.788 0.000  74 CPRS3 ~~ CPRS3 0.753 0.038 19.874 0.000  75 CPRS4 ~~ CPRS4 0.543 0.039 13.902 0.000  76 CPRS5 ~~ CPRS5 0.812 0.035 22.968 0.000  77 CPRS6 ~~ CPRS6 0.514 0.039 13.329 0.000  78 CPRS7 ~~ CPRS7 0.899 0.028 31.670 0.000  79 CPRS8 ~~ CPRS8 0.869 0.031 27.711 0.000  80 CPRS9 ~~ CPRS9 0.840 0.034 25.013 0.000  81 CPRS10 ~~ CPRS10 0.773 0.037 20.807 0.000  82 CPRS11 ~~ CPRS11 0.987 0.011 90.742 0.000  83 CPRS12 ~~ CPRS12 0.876 0.031 28.487 0.000  84 HE1 ~~ HE1 0.539 0.047 11.531 0.000  85 HE2 ~~ HE2 0.789 0.040 19.773 0.000  86 HE3 ~~ HE3 0.581 0.046 12.550 0.000  87 HE6 ~~ HE6 0.577 0.046 12.462 0.000  88 AN1 ~~ AN1 0.649 0.052 12.584 0.000  89 AN2 ~~ AN2 0.606 0.054 11.267 0.000  90 AN4 ~~ AN4 0.619 0.053 11.640 0.000  91 PT1 ~~ PT1 0.393 0.032 12.307 0.000  92 PT2 ~~ PT2 0.570 0.037 15.320 0.000  93 PT3 ~~ PT3 0.394 0.032 12.321 0.000  94 PT4 ~~ PT4 0.267 0.026 10.263 0.000  95 PT5 ~~ PT5 0.282 0.027 10.537 0.000  96 PT6 ~~ PT6 0.532 0.036 14.592 0.000  97 MT1 ~~ MT1 0.385 0.030 12.783 0.000  98 MT2 ~~ MT2 0.198 0.020 9.971 0.000  99 MT3 ~~ MT3 0.388 0.030 12.815 0.000  100 MT4 ~~ MT4 0.166 0.018 9.233 0.000  101 MT5 ~~ MT5 0.179 0.019 9.558 0.000  102 AW ~~ AW 1.000 0.000 NA NA  103 M ~~ M 1.000 0.000 NA NA  104 CPRS ~~ CPRS 1.000 0.000 NA NA  105 HE ~~ HE 1.000 0.000 NA NA  106 AN ~~ AN 1.000 0.000 NA NA  107 Prop ~~ Prop 1.000 0.000 NA NA  108 MT ~~ MT 1.000 0.000 NA NA |
| --- |
|  |
| \|  \| \| --- \| |

> fitMeasures(measM)

npar fmin **chisq df pvalue**

101.000 2.208 **2097.152 719.000 0.000**

baseline.chisq baseline.df baseline.pvalue **cfi tli**

9231.935 780.000 0.**000 0.837 0.823**

nnfi rfi nfi pnfi ifi

0.823 0.754 0.773 0.712 0.838

rni logl unrestricted.logl aic bic

0.837 -21215.851 -20167.275 42633.703 43054.198

ntotal bic2 **rmsea rmsea.ci.lower rmsea.ci.upper**

475.000 42733.639 **0.064 0.060 0.067**

rmsea.pvalue rmr rmr_nomean srmr srmr_bentler

0.000 0.073 0.073 0.084 0.084

srmr_bentler_nomean srmr_bollen srmr_bollen_nomean srmr_mplus srmr_mplus_nomean

0.084 0.084 0.084 0.084 0.084

cn_05 cn_01 gfi agfi pgfi

178.232 184.496 0.792 0.763 0.695

mfi ecvi

0.234 4.840

## ALTERNATE MEASUREMENT MODEL WITH PROPENSITY AS HIGHER-ORDER VARIABLE

> standardizedSolution(altMeas)

lhs op rhs est.std se z pvalue

1 AW =~ AICP1 0.797 0.022 36.581 0.000

2 AW =~ AICP2 0.805 0.021 37.612 0.000

3 AW =~ AICP3 0.652 0.030 21.516 0.000

4 AW =~ AICP4 0.683 0.029 23.948 0.000

5 AW =~ AICP6 0.628 0.032 19.835 0.000

6 M =~ AICP5 0.422 0.044 9.646 0.000

7 M =~ AICP7 0.647 0.034 18.920 0.000

8 M =~ AICP8 0.654 0.034 19.323 0.000

9 M =~ AICP9 0.757 0.030 25.627 0.000

10 M =~ AICP10 0.669 0.033 20.141 0.000

11 CPRS =~ CPRS1 0.682 0.029 23.368 0.000

12 CPRS =~ CPRS2 0.654 0.031 21.280 0.000

13 CPRS =~ CPRS3 0.513 0.038 13.555 0.000

14 CPRS =~ CPRS4 0.673 0.030 22.651 0.000

15 CPRS =~ CPRS5 0.441 0.041 10.793 0.000

16 CPRS =~ CPRS6 0.682 0.029 23.420 0.000

17 CPRS =~ CPRS7 0.313 0.045 6.912 0.000

18 CPRS =~ CPRS8 0.344 0.044 7.757 0.000

19 CPRS =~ CPRS9 0.397 0.043 9.332 0.000

20 CPRS =~ CPRS10 0.476 0.039 12.060 0.000

21 CPRS =~ CPRS11 0.126 0.049 2.579 0.010

22 CPRS =~ CPRS12 0.350 0.044 7.943 0.000

23 HE =~ HE1 0.637 0.039 16.295 0.000

24 HE =~ HE2 0.506 0.044 11.621 0.000

25 HE =~ HE3 0.700 0.037 18.665 0.000

26 HE =~ HE6 0.589 0.041 14.489 0.000

27 AN =~ AN1 0.703 0.052 13.447 0.000

28 AN =~ AN2 0.603 0.050 12.146 0.000

29 AN =~ AN4 0.542 0.049 11.173 0.000

30 Prop =~ PT1 0.777 0.021 37.883 0.000

31 Prop =~ PT2 0.661 0.028 23.593 0.000

32 Prop =~ PT3 0.781 0.020 38.436 0.000

33 Prop =~ PT4 0.849 0.016 54.530 0.000

34 Prop =~ PT5 0.842 0.016 52.473 0.000

35 Prop =~ PT6 0.675 0.027 24.888 0.000

36 MT =~ MT1 0.784 0.019 40.743 0.000

37 MT =~ MT2 0.896 0.011 80.910 0.000

38 MT =~ MT3 0.784 0.019 40.742 0.000

39 MT =~ MT4 0.914 0.010 93.097 0.000

40 MT =~ MT5 0.906 0.010 87.281 0.000

**41 Prop =~ AW 0.689 0.030 22.613 0.000 AW, HE and CPRS load pretty well.**

**42 Prop =~ M 0.209 0.052 4.046 0.000 M, AN, and MT have low loadings.**

**43 Prop =~ CPRS 0.754 0.027 27.632 0.000**

**44 Prop =~ HE 0.419 0.049 8.562 0.000**

**45 Prop =~ AN -0.037 0.059 -0.628 0.530**

**46 Prop =~ MT 0.105 0.049 2.154 0.031**

47 AICP1 ~~ AICP1 0.364 0.035 10.475 0.000

48 AICP2 ~~ AICP2 0.353 0.034 10.250 0.000

49 AICP3 ~~ AICP3 0.575 0.040 14.538 0.000

50 AICP4 ~~ AICP4 0.533 0.039 13.673 0.000

51 AICP6 ~~ AICP6 0.606 0.040 15.241 0.000

52 AICP5 ~~ AICP5 0.822 0.037 22.244 0.000

53 AICP7 ~~ AICP7 0.582 0.044 13.156 0.000

54 AICP8 ~~ AICP8 0.572 0.044 12.923 0.000

55 AICP9 ~~ AICP9 0.428 0.045 9.574 0.000

56 AICP10 ~~ AICP10 0.553 0.044 12.463 0.000

57 CPRS1 ~~ CPRS1 0.535 0.040 13.455 0.000

58 CPRS2 ~~ CPRS2 0.572 0.040 14.235 0.000

59 CPRS3 ~~ CPRS3 0.737 0.039 18.948 0.000

60 CPRS4 ~~ CPRS4 0.548 0.040 13.710 0.000

61 CPRS5 ~~ CPRS5 0.805 0.036 22.292 0.000

62 CPRS6 ~~ CPRS6 0.534 0.040 13.437 0.000

63 CPRS7 ~~ CPRS7 0.902 0.028 31.890 0.000

64 CPRS8 ~~ CPRS8 0.882 0.030 28.929 0.000

65 CPRS9 ~~ CPRS9 0.842 0.034 24.916 0.000

66 CPRS10 ~~ CPRS10 0.773 0.038 20.559 0.000

67 CPRS11 ~~ CPRS11 0.984 0.012 79.735 0.000

68 CPRS12 ~~ CPRS12 0.877 0.031 28.368 0.000

69 HE1 ~~ HE1 0.594 0.050 11.915 0.000

70 HE2 ~~ HE2 0.744 0.044 16.853 0.000

71 HE3 ~~ HE3 0.510 0.052 9.721 0.000

72 HE6 ~~ HE6 0.653 0.048 13.657 0.000

73 AN1 ~~ AN1 0.506 0.073 6.899 0.000

74 AN2 ~~ AN2 0.636 0.060 10.613 0.000

75 AN4 ~~ AN4 0.706 0.053 13.414 0.000

76 PT1 ~~ PT1 0.396 0.032 12.398 0.000

77 PT2 ~~ PT2 0.563 0.037 15.228 0.000

78 PT3 ~~ PT3 0.391 0.032 12.324 0.000

79 PT4 ~~ PT4 0.280 0.026 10.577 0.000

80 PT5 ~~ PT5 0.291 0.027 10.775 0.000

81 PT6 ~~ PT6 0.544 0.037 14.850 0.000

82 MT1 ~~ MT1 0.386 0.030 12.786 0.000

83 MT2 ~~ MT2 0.198 0.020 9.966 0.000

84 MT3 ~~ MT3 0.386 0.030 12.786 0.000

85 MT4 ~~ MT4 0.165 0.018 9.218 0.000

86 MT5 ~~ MT5 0.180 0.019 9.580 0.000

87 AW ~~ AW 0.525 0.042 12.485 0.000

88 M ~~ M 0.956 0.022 44.109 0.000

89 CPRS ~~ CPRS 0.431 0.041 10.473 0.000

90 HE ~~ HE 0.825 0.041 20.134 0.000

91 AN ~~ AN 0.999 0.004 230.563 0.000

92 Prop ~~ Prop 1.000 0.000 NA NA

93 MT ~~ MT 0.989 0.010 96.644 0.000

fitMeasures(altMeas)

npar fmin chisq df pvalue

86.000 2.447 2324.312 734.000 0.000

baseline.chisq baseline.df baseline.pvalue cfi tli

9231.935 780.000 0.000 0.812 0.800

nnfi rfi nfi pnfi ifi

0.800 0.732 0.748 0.704 0.813

rni logl unrestricted.logl aic bic

0.812 -21329.431 -20167.275 42830.862 43188.907

ntotal bic2 rmsea rmsea.ci.lower rmsea.ci.upper

475.000 42915.956 0.068 0.064 0.071

rmsea.pvalue rmr rmr_nomean srmr srmr_bentler

0.000 0.080 0.080 0.093 0.093

srmr_bentler_nomean srmr_bollen srmr_bollen_nomean srmr_mplus srmr_mplus_nomean

0.093 0.093 0.093 0.093 0.093

cn_05 cn_01 gfi agfi pgfi

164.109 169.815 0.764 0.736 0.684

mfi ecvi

0.187 5.255

# Change in df = 15, change in X2 = -227.16

# Change in X2 is significant at *p* < .01 – fit of this model is significantly worse than the basic measurement model.

## ANOTHER ALTERNATE MODEL ##


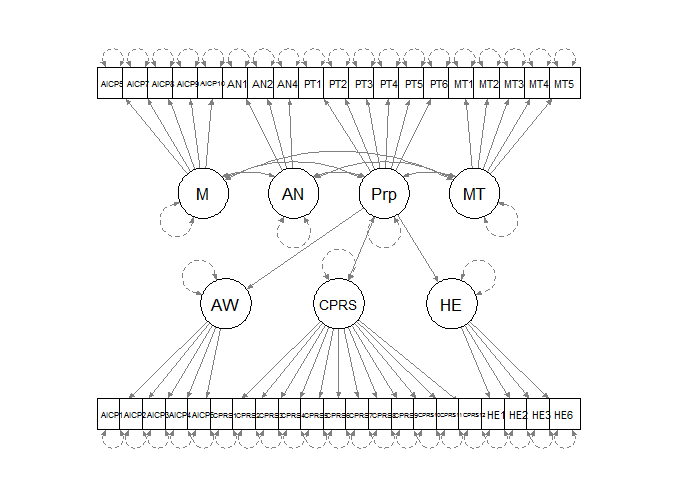


| > standardizedSolution(altMod2)  lhs op rhs est.std se z pvalue  1 AW =~ AICP1 0.797 0.022 36.577 0.000  2 AW =~ AICP2 0.805 0.021 37.622 0.000  3 AW =~ AICP3 0.652 0.030 21.518 0.000  4 AW =~ AICP4 0.683 0.029 23.946 0.000  5 AW =~ AICP6 0.628 0.032 19.832 0.000  6 M =~ AICP5 0.418 0.044 9.538 0.000  7 M =~ AICP7 0.649 0.034 19.094 0.000  8 M =~ AICP8 0.649 0.034 19.081 0.000  9 M =~ AICP9 0.758 0.029 25.758 0.000  10 M =~ AICP10 0.671 0.033 20.313 0.000  11 CPRS =~ CPRS1 0.682 0.029 23.369 0.000  12 CPRS =~ CPRS2 0.654 0.031 21.281 0.000  13 CPRS =~ CPRS3 0.513 0.038 13.553 0.000  14 CPRS =~ CPRS4 0.673 0.030 22.652 0.000  15 CPRS =~ CPRS5 0.441 0.041 10.791 0.000  16 CPRS =~ CPRS6 0.682 0.029 23.428 0.000  17 CPRS =~ CPRS7 0.313 0.045 6.915 0.000  18 CPRS =~ CPRS8 0.344 0.044 7.761 0.000  19 CPRS =~ CPRS9 0.397 0.043 9.333 0.000  20 CPRS =~ CPRS10 0.476 0.039 12.057 0.000  21 CPRS =~ CPRS11 0.126 0.049 2.576 0.010  22 CPRS =~ CPRS12 0.350 0.044 7.941 0.000  23 HE =~ HE1 0.637 0.039 16.299 0.000  24 HE =~ HE2 0.506 0.044 11.613 0.000  25 HE =~ HE3 0.700 0.038 18.659 0.000  26 HE =~ HE6 0.589 0.041 14.492 0.000  27 AN =~ AN1 0.697 0.052 13.504 0.000  28 AN =~ AN2 0.607 0.049 12.274 0.000  29 AN =~ AN4 0.544 0.048 11.249 0.000  30 Prop =~ PT1 0.778 0.021 37.905 0.000  31 Prop =~ PT2 0.661 0.028 23.627 0.000  32 Prop =~ PT3 0.781 0.020 38.445 0.000  33 Prop =~ PT4 0.849 0.016 54.493 0.000  34 Prop =~ PT5 0.842 0.016 52.446 0.000  35 Prop =~ PT6 0.675 0.027 24.866 0.000  36 MT =~ MT1 0.784 0.019 40.733 0.000  37 MT =~ MT2 0.896 0.011 80.794 0.000  38 MT =~ MT3 0.784 0.019 40.711 0.000  39 MT =~ MT4 0.914 0.010 93.119 0.000  40 MT =~ MT5 0.906 0.010 87.507 0.000  **41 Prop =~ AW 0.689 0.030 22.617 0.000**  **42 Prop =~ HE 0.418 0.049 8.536 0.000**  **43 Prop =~ CPRS 0.754 0.027 27.655 0.000**  **44 M ~~ Prop 0.209 0.052 4.040 0.000**  **45 AN ~~ Prop -0.040 0.059 -0.677 0.498**  **46 Prop ~~ MT 0.104 0.049 2.129 0.033**  **47 M ~~ AN 0.074 0.063 1.166 0.244**  **48 M ~~ MT 0.101 0.053 1.922 0.055**  **49 AN ~~ MT 0.035 0.058 0.611 0.541**  50 AICP1 ~~ AICP1 0.364 0.035 10.476 0.000  51 AICP2 ~~ AICP2 0.353 0.034 10.248 0.000  52 AICP3 ~~ AICP3 0.575 0.040 14.537 0.000  53 AICP4 ~~ AICP4 0.533 0.039 13.674 0.000  54 AICP6 ~~ AICP6 0.606 0.040 15.242 0.000  55 AICP5 ~~ AICP5 0.825 0.037 22.476 0.000  56 AICP7 ~~ AICP7 0.578 0.044 13.086 0.000  57 AICP8 ~~ AICP8 0.578 0.044 13.093 0.000  58 AICP9 ~~ AICP9 0.426 0.045 9.557 0.000  59 AICP10 ~~ AICP10 0.550 0.044 12.402 0.000  60 CPRS1 ~~ CPRS1 0.535 0.040 13.456 0.000  61 CPRS2 ~~ CPRS2 0.572 0.040 14.236 0.000  62 CPRS3 ~~ CPRS3 0.737 0.039 18.951 0.000  63 CPRS4 ~~ CPRS4 0.548 0.040 13.711 0.000  64 CPRS5 ~~ CPRS5 0.805 0.036 22.296 0.000  65 CPRS6 ~~ CPRS6 0.534 0.040 13.435 0.000  66 CPRS7 ~~ CPRS7 0.902 0.028 31.877 0.000  67 CPRS8 ~~ CPRS8 0.882 0.030 28.919 0.000  68 CPRS9 ~~ CPRS9 0.842 0.034 24.914 0.000  69 CPRS10 ~~ CPRS10 0.773 0.038 20.564 0.000  70 CPRS11 ~~ CPRS11 0.984 0.012 79.821 0.000  71 CPRS12 ~~ CPRS12 0.877 0.031 28.374 0.000  72 HE1 ~~ HE1 0.594 0.050 11.907 0.000  73 HE2 ~~ HE2 0.744 0.044 16.861 0.000  74 HE3 ~~ HE3 0.510 0.052 9.721 0.000  75 HE6 ~~ HE6 0.653 0.048 13.651 0.000  76 AN1 ~~ AN1 0.514 0.072 7.133 0.000  77 AN2 ~~ AN2 0.632 0.060 10.526 0.000  78 AN4 ~~ AN4 0.704 0.053 13.372 0.000  79 PT1 ~~ PT1 0.395 0.032 12.394 0.000  80 PT2 ~~ PT2 0.563 0.037 15.217 0.000  81 PT3 ~~ PT3 0.391 0.032 12.322 0.000  82 PT4 ~~ PT4 0.280 0.026 10.580 0.000  83 PT5 ~~ PT5 0.291 0.027 10.777 0.000  84 PT6 ~~ PT6 0.544 0.037 14.856 0.000  85 MT1 ~~ MT1 0.386 0.030 12.788 0.000  86 MT2 ~~ MT2 0.198 0.020 9.976 0.000  87 MT3 ~~ MT3 0.386 0.030 12.790 0.000  88 MT4 ~~ MT4 0.165 0.018 9.220 0.000  89 MT5 ~~ MT5 0.179 0.019 9.569 0.000  90 AW ~~ AW 0.525 0.042 12.483 0.000  91 M ~~ M 1.000 0.000 NA NA  92 CPRS ~~ CPRS 0.431 0.041 10.467 0.000  93 HE ~~ HE 0.825 0.041 20.186 0.000  94 AN ~~ AN 1.000 0.000 NA NA  95 Prop ~~ Prop 1.000 0.000 NA NA  96 MT ~~ MT 1.000 0.000 NA NA |
| --- |
|  |
| \| > fitMeasures(altMod2)  npar fmin chisq df  89.000 2.442 **2319.940 731.000**  pvalue baseline.chisq baseline.df baseline.pvalue  0.000 9231.935 780.000 0.000  cfi tli nnfi rfi  **0.812 0.799** 0.799 0.732  nfi pnfi ifi rni  0.749 0.702 0.813 0.812  logl unrestricted.logl aic bic  -21327.245 -20167.275 42832.490 43203.025  ntotal bic2 rmsea rmsea.ci.lower  475.000 42920.**553 0.068 0.065**  rmsea.ci.upper rmsea.pvalue rmr rmr_nomean  **0.071 0.000** 0.079 0.079  srmr srmr_bentler srmr_bentler_nomean srmr_bollen  0.093 0.093 0.093 0.093  srmr_bollen_nomean srmr_mplus srmr_mplus_nomean cn_05  0.093 0.093 0.093 163.775  cn_01 gfi agfi pgfi  169.482 0.765 0.737 0.682  mfi ecvi  0.188 5.259 \| \| --- \| |

| > standardizedSolution(altMod2)  lhs op rhs est.std se z pvalue  1 AW =~ AICP1 0.797 0.022 36.577 0.000  2 AW =~ AICP2 0.805 0.021 37.622 0.000  3 AW =~ AICP3 0.652 0.030 21.518 0.000  4 AW =~ AICP4 0.683 0.029 23.946 0.000  5 AW =~ AICP6 0.628 0.032 19.832 0.000  6 M =~ AICP5 0.418 0.044 9.538 0.000  7 M =~ AICP7 0.649 0.034 19.094 0.000  8 M =~ AICP8 0.649 0.034 19.081 0.000  9 M =~ AICP9 0.758 0.029 25.758 0.000  10 M =~ AICP10 0.671 0.033 20.313 0.000  11 CPRS =~ CPRS1 0.682 0.029 23.369 0.000  12 CPRS =~ CPRS2 0.654 0.031 21.281 0.000  13 CPRS =~ CPRS3 0.513 0.038 13.553 0.000  14 CPRS =~ CPRS4 0.673 0.030 22.652 0.000  15 CPRS =~ CPRS5 0.441 0.041 10.791 0.000  16 CPRS =~ CPRS6 0.682 0.029 23.428 0.000  17 CPRS =~ CPRS7 0.313 0.045 6.915 0.000  18 CPRS =~ CPRS8 0.344 0.044 7.761 0.000  19 CPRS =~ CPRS9 0.397 0.043 9.333 0.000  20 CPRS =~ CPRS10 0.476 0.039 12.057 0.000  21 CPRS =~ CPRS11 0.126 0.049 2.576 0.010  22 CPRS =~ CPRS12 0.350 0.044 7.941 0.000  23 HE =~ HE1 0.637 0.039 16.299 0.000  24 HE =~ HE2 0.506 0.044 11.613 0.000  25 HE =~ HE3 0.700 0.038 18.659 0.000  26 HE =~ HE6 0.589 0.041 14.492 0.000  27 AN =~ AN1 0.697 0.052 13.504 0.000  28 AN =~ AN2 0.607 0.049 12.274 0.000  29 AN =~ AN4 0.544 0.048 11.249 0.000  30 Prop =~ PT1 0.778 0.021 37.905 0.000  31 Prop =~ PT2 0.661 0.028 23.627 0.000  32 Prop =~ PT3 0.781 0.020 38.445 0.000  33 Prop =~ PT4 0.849 0.016 54.493 0.000  34 Prop =~ PT5 0.842 0.016 52.446 0.000  35 Prop =~ PT6 0.675 0.027 24.866 0.000  36 MT =~ MT1 0.784 0.019 40.733 0.000  37 MT =~ MT2 0.896 0.011 80.794 0.000  38 MT =~ MT3 0.784 0.019 40.711 0.000  39 MT =~ MT4 0.914 0.010 93.119 0.000  40 MT =~ MT5 0.906 0.010 87.507 0.000  41 Prop =~ AW 0.689 0.030 22.617 0.000  42 Prop =~ HE 0.418 0.049 8.536 0.000  43 Prop =~ CPRS 0.754 0.027 27.655 0.000  44 M ~~ Prop 0.209 0.052 4.040 0.000  45 AN ~~ Prop -0.040 0.059 -0.677 0.498  46 Prop ~~ MT 0.104 0.049 2.129 0.033  47 M ~~ AN 0.074 0.063 1.166 0.244  48 M ~~ MT 0.101 0.053 1.922 0.055  49 AN ~~ MT 0.035 0.058 0.611 0.541  50 AICP1 ~~ AICP1 0.364 0.035 10.476 0.000  51 AICP2 ~~ AICP2 0.353 0.034 10.248 0.000  52 AICP3 ~~ AICP3 0.575 0.040 14.537 0.000  53 AICP4 ~~ AICP4 0.533 0.039 13.674 0.000  54 AICP6 ~~ AICP6 0.606 0.040 15.242 0.000  55 AICP5 ~~ AICP5 0.825 0.037 22.476 0.000  56 AICP7 ~~ AICP7 0.578 0.044 13.086 0.000  57 AICP8 ~~ AICP8 0.578 0.044 13.093 0.000  58 AICP9 ~~ AICP9 0.426 0.045 9.557 0.000  59 AICP10 ~~ AICP10 0.550 0.044 12.402 0.000  60 CPRS1 ~~ CPRS1 0.535 0.040 13.456 0.000  61 CPRS2 ~~ CPRS2 0.572 0.040 14.236 0.000  62 CPRS3 ~~ CPRS3 0.737 0.039 18.951 0.000  63 CPRS4 ~~ CPRS4 0.548 0.040 13.711 0.000  64 CPRS5 ~~ CPRS5 0.805 0.036 22.296 0.000  65 CPRS6 ~~ CPRS6 0.534 0.040 13.435 0.000  66 CPRS7 ~~ CPRS7 0.902 0.028 31.877 0.000  67 CPRS8 ~~ CPRS8 0.882 0.030 28.919 0.000  68 CPRS9 ~~ CPRS9 0.842 0.034 24.914 0.000  69 CPRS10 ~~ CPRS10 0.773 0.038 20.564 0.000  70 CPRS11 ~~ CPRS11 0.984 0.012 79.821 0.000  71 CPRS12 ~~ CPRS12 0.877 0.031 28.374 0.000  72 HE1 ~~ HE1 0.594 0.050 11.907 0.000  73 HE2 ~~ HE2 0.744 0.044 16.861 0.000  74 HE3 ~~ HE3 0.510 0.052 9.721 0.000  75 HE6 ~~ HE6 0.653 0.048 13.651 0.000  76 AN1 ~~ AN1 0.514 0.072 7.133 0.000  77 AN2 ~~ AN2 0.632 0.060 10.526 0.000  78 AN4 ~~ AN4 0.704 0.053 13.372 0.000  79 PT1 ~~ PT1 0.395 0.032 12.394 0.000  80 PT2 ~~ PT2 0.563 0.037 15.217 0.000  81 PT3 ~~ PT3 0.391 0.032 12.322 0.000  82 PT4 ~~ PT4 0.280 0.026 10.580 0.000  83 PT5 ~~ PT5 0.291 0.027 10.777 0.000  84 PT6 ~~ PT6 0.544 0.037 14.856 0.000  85 MT1 ~~ MT1 0.386 0.030 12.788 0.000  86 MT2 ~~ MT2 0.198 0.020 9.976 0.000  87 MT3 ~~ MT3 0.386 0.030 12.790 0.000  88 MT4 ~~ MT4 0.165 0.018 9.220 0.000  89 MT5 ~~ MT5 0.179 0.019 9.569 0.000  90 AW ~~ AW 0.525 0.042 12.483 0.000  91 M ~~ M 1.000 0.000 NA NA  92 CPRS ~~ CPRS 0.431 0.041 10.467 0.000  93 HE ~~ HE 0.825 0.041 20.186 0.000  94 AN ~~ AN 1.000 0.000 NA NA  95 Prop ~~ Prop 1.000 0.000 NA NA  96 MT ~~ MT 1.000 0.000 NA NA |
| --- |
|  |
| \| > \| \| --- \| |

| > standardizedSolution(altMod2)  lhs op rhs est.std se z pvalue  1 AW =~ AICP1 0.797 0.022 36.577 0.000  2 AW =~ AICP2 0.805 0.021 37.622 0.000  3 AW =~ AICP3 0.652 0.030 21.518 0.000  4 AW =~ AICP4 0.683 0.029 23.946 0.000  5 AW =~ AICP6 0.628 0.032 19.832 0.000  6 M =~ AICP5 0.418 0.044 9.538 0.000  7 M =~ AICP7 0.649 0.034 19.094 0.000  8 M =~ AICP8 0.649 0.034 19.081 0.000  9 M =~ AICP9 0.758 0.029 25.758 0.000  10 M =~ AICP10 0.671 0.033 20.313 0.000  11 CPRS =~ CPRS1 0.682 0.029 23.369 0.000  12 CPRS =~ CPRS2 0.654 0.031 21.281 0.000  13 CPRS =~ CPRS3 0.513 0.038 13.553 0.000  14 CPRS =~ CPRS4 0.673 0.030 22.652 0.000  15 CPRS =~ CPRS5 0.441 0.041 10.791 0.000  16 CPRS =~ CPRS6 0.682 0.029 23.428 0.000  17 CPRS =~ CPRS7 0.313 0.045 6.915 0.000  18 CPRS =~ CPRS8 0.344 0.044 7.761 0.000  19 CPRS =~ CPRS9 0.397 0.043 9.333 0.000  20 CPRS =~ CPRS10 0.476 0.039 12.057 0.000  21 CPRS =~ CPRS11 0.126 0.049 2.576 0.010  22 CPRS =~ CPRS12 0.350 0.044 7.941 0.000  23 HE =~ HE1 0.637 0.039 16.299 0.000  24 HE =~ HE2 0.506 0.044 11.613 0.000  25 HE =~ HE3 0.700 0.038 18.659 0.000  26 HE =~ HE6 0.589 0.041 14.492 0.000  27 AN =~ AN1 0.697 0.052 13.504 0.000  28 AN =~ AN2 0.607 0.049 12.274 0.000  29 AN =~ AN4 0.544 0.048 11.249 0.000  30 Prop =~ PT1 0.778 0.021 37.905 0.000  31 Prop =~ PT2 0.661 0.028 23.627 0.000  32 Prop =~ PT3 0.781 0.020 38.445 0.000  33 Prop =~ PT4 0.849 0.016 54.493 0.000  34 Prop =~ PT5 0.842 0.016 52.446 0.000  35 Prop =~ PT6 0.675 0.027 24.866 0.000  36 MT =~ MT1 0.784 0.019 40.733 0.000  37 MT =~ MT2 0.896 0.011 80.794 0.000  38 MT =~ MT3 0.784 0.019 40.711 0.000  39 MT =~ MT4 0.914 0.010 93.119 0.000  40 MT =~ MT5 0.906 0.010 87.507 0.000  41 Prop =~ AW 0.689 0.030 22.617 0.000  42 Prop =~ HE 0.418 0.049 8.536 0.000  43 Prop =~ CPRS 0.754 0.027 27.655 0.000  44 M ~~ Prop 0.209 0.052 4.040 0.000  45 AN ~~ Prop -0.040 0.059 -0.677 0.498  46 Prop ~~ MT 0.104 0.049 2.129 0.033  47 M ~~ AN 0.074 0.063 1.166 0.244  48 M ~~ MT 0.101 0.053 1.922 0.055  49 AN ~~ MT 0.035 0.058 0.611 0.541  50 AICP1 ~~ AICP1 0.364 0.035 10.476 0.000  51 AICP2 ~~ AICP2 0.353 0.034 10.248 0.000  52 AICP3 ~~ AICP3 0.575 0.040 14.537 0.000  53 AICP4 ~~ AICP4 0.533 0.039 13.674 0.000  54 AICP6 ~~ AICP6 0.606 0.040 15.242 0.000  55 AICP5 ~~ AICP5 0.825 0.037 22.476 0.000  56 AICP7 ~~ AICP7 0.578 0.044 13.086 0.000  57 AICP8 ~~ AICP8 0.578 0.044 13.093 0.000  58 AICP9 ~~ AICP9 0.426 0.045 9.557 0.000  59 AICP10 ~~ AICP10 0.550 0.044 12.402 0.000  60 CPRS1 ~~ CPRS1 0.535 0.040 13.456 0.000  61 CPRS2 ~~ CPRS2 0.572 0.040 14.236 0.000  62 CPRS3 ~~ CPRS3 0.737 0.039 18.951 0.000  63 CPRS4 ~~ CPRS4 0.548 0.040 13.711 0.000  64 CPRS5 ~~ CPRS5 0.805 0.036 22.296 0.000  65 CPRS6 ~~ CPRS6 0.534 0.040 13.435 0.000  66 CPRS7 ~~ CPRS7 0.902 0.028 31.877 0.000  67 CPRS8 ~~ CPRS8 0.882 0.030 28.919 0.000  68 CPRS9 ~~ CPRS9 0.842 0.034 24.914 0.000  69 CPRS10 ~~ CPRS10 0.773 0.038 20.564 0.000  70 CPRS11 ~~ CPRS11 0.984 0.012 79.821 0.000  71 CPRS12 ~~ CPRS12 0.877 0.031 28.374 0.000  72 HE1 ~~ HE1 0.594 0.050 11.907 0.000  73 HE2 ~~ HE2 0.744 0.044 16.861 0.000  74 HE3 ~~ HE3 0.510 0.052 9.721 0.000  75 HE6 ~~ HE6 0.653 0.048 13.651 0.000  76 AN1 ~~ AN1 0.514 0.072 7.133 0.000  77 AN2 ~~ AN2 0.632 0.060 10.526 0.000  78 AN4 ~~ AN4 0.704 0.053 13.372 0.000  79 PT1 ~~ PT1 0.395 0.032 12.394 0.000  80 PT2 ~~ PT2 0.563 0.037 15.217 0.000  81 PT3 ~~ PT3 0.391 0.032 12.322 0.000  82 PT4 ~~ PT4 0.280 0.026 10.580 0.000  83 PT5 ~~ PT5 0.291 0.027 10.777 0.000  84 PT6 ~~ PT6 0.544 0.037 14.856 0.000  85 MT1 ~~ MT1 0.386 0.030 12.788 0.000  86 MT2 ~~ MT2 0.198 0.020 9.976 0.000  87 MT3 ~~ MT3 0.386 0.030 12.790 0.000  88 MT4 ~~ MT4 0.165 0.018 9.220 0.000  89 MT5 ~~ MT5 0.179 0.019 9.569 0.000  90 AW ~~ AW 0.525 0.042 12.483 0.000  91 M ~~ M 1.000 0.000 NA NA  92 CPRS ~~ CPRS 0.431 0.041 10.467 0.000  93 HE ~~ HE 0.825 0.041 20.186 0.000  94 AN ~~ AN 1.000 0.000 NA NA  95 Prop ~~ Prop 1.000 0.000 NA NA  96 MT ~~ MT 1.000 0.000 NA NA |
| --- |
|  |
| \| > \| \| --- \| |
